# Transcriptome and Proteomics Analysis of Wheat Seedling Roots Reveals That Increasing NH_4_^+^/NO_3_^–^ Ratio Induced Root Lignification and Reduced Nitrogen Utilization

**DOI:** 10.3389/fpls.2021.797260

**Published:** 2022-01-13

**Authors:** Dongqing Yang, Jihao Zhao, Chen Bi, Liuyin Li, Zhenlin Wang

**Affiliations:** State Key Laboratory of Crop Biology, College of Agronomy, Shandong Agricultural University, Taian, China

**Keywords:** root length, ROS, glutathione transferase, lignin, nitrogen transport, transcriptome, proteomic

## Abstract

Wheat growth and nitrogen (N) uptake gradually decrease in response to high NH_4_^+^/NO_3_^–^ ratio. However, the mechanisms underlying the response of wheat seedling roots to changes in NH_4_^+^/NO_3_^–^ ratio remain unclear. In this study, we investigated wheat growth, transcriptome, and proteome profiles of roots in response to increasing NH_4_^+^/NO_3_^–^ ratios (N_*a*_: 100/0; N_*r*1_: 75/25, N_*r*2_: 50/50, N_*r*3_: 25/75, and N_*n*_: 0/100). High NH_4_^+^/NO_3_^–^ ratio significantly reduced leaf relative chlorophyll content, Fv/Fm, and ΦII values. Both total root length and specific root length decreased with increasing NH_4_^+^/NO_3_^–^ ratios. Moreover, the rise in NH_4_^+^/NO_3_^–^ ratio significantly promoted O_2_^–^ production. Furthermore, transcriptome sequencing and tandem mass tag-based quantitative proteome analyses identified 14,376 differentially expressed genes (DEGs) and 1,819 differentially expressed proteins (DEPs). The Kyoto Encyclopedia of Genes and Genomes (KEGG) pathway enrichment analysis indicated that glutathione metabolism and phenylpropanoid biosynthesis were the main two shared enriched pathways across ratio comparisons. Upregulated DEGs and DEPs involving glutathione S-transferases may contribute to the prevention of oxidative stress. An increment in the NH_4_^+^/NO_3_^–^ ratio induced the expression of genes and proteins involved in lignin biosynthesis, which increased root lignin content. Additionally, phylogenetic tree analysis showed that both A0A3B6NPP6 and A0A3B6LM09 belong to the cinnamyl-alcohol dehydrogenase subfamily. Fifteen downregulated DEGs were identified as high-affinity nitrate transporters or nitrate transporters. Upregulated *TraesCS3D02G344800* and *TraesCS3A02G350800* were involved in ammonium transport. Downregulated A0A3B6Q9B3 is involved in nitrate transport, whereas A0A3B6PQS3 is a ferredoxin-nitrite reductase. This may explain why an increase in the NH_4_^+^/NO_3_^–^ ratio significantly reduced root NO_3_^–^-N content but increased NH_4_^+^-N content. Overall, these results demonstrated that increasing the NH_4_^+^/NO_3_^–^ ratio at the seedling stage induced the accumulation of reactive oxygen species, which in turn enhanced root glutathione metabolism and lignification, thereby resulting in increased root oxidative tolerance at the cost of reducing nitrate transport and utilization, which reduced leaf photosynthetic capacity and, ultimately, plant biomass accumulation.

## Introduction

Wheat (*Triticum aestivum* L.) is one of the most important cereal foods worldwide. The tremendous increase in wheat crop productivity over the past several decades has made China the largest wheat consumer and producer in the world (Xu et al., 2013), mainly because of the substantial increase in the use of synthetic fertilizers, specifically, nitrogen (N) (Zhu and Chen, 2002; Yuan and Peng, 2017).

Plants have evolved sophisticated root regulatory mechanisms and N transport systems for soil N uptake (Luo et al., 2020). Although previous studies showed that urea was directly absorbed by urea transporters, such as DUR3 and MIP (Wang et al., 2012; Liu et al., 2015), it is widely accepted that plants absorb mainly ammonium or nitrate generated by microbial conversion or *via* their soil application as fertilizers (Witte, 2011). Two soil nitrate and ammonium uptake systems have been identified in higher plants, namely, the high-affinity transport system (HATS) and the low-affinity transport system (LATS) (Kiba and Krapp, 2016; Gojon, 2017; Plett et al., 2018). NRT1 and NRT2 are operated by two families of nitrate transporters, whereas members of the ammonium transporter (AMT) family mediate ammonium transport (Kraiser et al., 2011) and are involved in diverse aspects of plant growth and development. Although N transporters have also been reported to have specific effects on lateral root initiation (Remans et al., 2006; Motte et al., 2019), there are few reports on the effects of different NH_4_^+^/NO_3_^–^ ratios on changes in N transport and uptake and metabolism in roots of young wheat seedlings.

Farmers apply excessive amounts of N fertilizers, such as urea, ammonium bicarbonate, and ammonium nitrate, simply following the conventional concept of “the more fertilizer, the higher yield.” However, studies have shown that grain yield does not increase accordingly with the excess of applied N (Ju et al., 2009; Cui et al., 2010; Liu et al., 2016). Indeed, although large amounts of N fertilizer are regularly applied as base fertilizer to increase the NH_4_^+^-N concentration and, thus, change the ratio of NH_4_^+^ and NO_3_^–^ in the soil (Husain et al., 2019; Kirschke et al., 2019; Wang et al., 2020), a high-NH_4_^+^ concentration may be a disadvantage for plant growth. Wheat was shown to have a specific preference for NO_3_^–^, and in fact, showed toxicity symptoms under high NH_4_^+^ concentration (Imran et al., 2019). Consistently, plant biomass was significantly reduced when N fertilizer was supplied as NH_4_^+^ (Wang et al., 2016a), likely because NH_4_^+^ reduced plant photosynthetic capacity due to damage to the electron transport chain (Wang et al., 2019). On the other hand, NH_4_^+^-N promoted over-accumulation of glutamate, which can inhibit pyruvate kinase activity, causing a reduction in tricarboxylic acid flux (Wang et al., 2020). Furthermore, NH_4_^+^-N caused oxidative stress due to the enhanced generation of reactive oxygen species (ROS), resulting in root growth inhibition (Liu et al., 2021). Fortunately, plants possess defense mechanisms to cope with oxidative stress, whereby cells are protected against potential oxidative damage caused by ROS.

Glutathione metabolism is one of the defense mechanisms that play a vital role in plant resistance to oxidative stress (Gong et al., 2018). Plant glutathione and glutathione S-transferases (GSTs) are widely involved in ROS detoxification (Zechmann, 2014; Ding et al., 2017). As a potent antioxidant, glutathione can control cellular ROS levels (Gill et al., 2013). GSTs catalyze the nucleophilic fusion of reduced glutathione with electrophilic and hydrophobic toxic molecules generated under stress to convert them into non-toxic soluble conjugates (Tiwari et al., 2016). Lignin deposition is another defense mechanism that responds to a wide range of abiotic stress conditions (Cesarino, 2019) to which plants are often exposed during their life cycle (Moura et al., 2010). For example, salt and drought stress upregulated the expression of the *phenylalanine ammonia-lyase* (*PAL*), *4-coumarate-CoA ligase* (*4CL*), *cinnamoyl-CoA reductase* (*CCR*), and *cinnamyl alcohol dehydrogenase* (*CAD*) genes, resulting in an increase in lignin biosynthesis, enhancing stress resistance (Liu et al., 2018; Xu et al., 2020). Although glutathione metabolism and lignin biosynthesis have been reported during plant development and under abiotic stress conditions, limited information is available regarding the involvement of these two defense strategies in wheat roots under NH_4_^+^ stress. In particular, the mechanisms underlying the responses of glutathione metabolism and lignin biosynthesis in wheat root growth to different NH_4_^+^/NO_3_^–^ ratios are still far from being clearly understood. Therefore, the objectives of the present study were to (i) identify the effects of different NH_4_^+^/NO_3_^–^ ratios on plant and root growth at the seedling stage, (ii) investigate the wheat root transcriptome and proteome profiles related to glutathione metabolism and lignin biosynthesis in response to different NH_4_^+^/NO_3_^–^ ratios, and (iii) provide new insights into the wheat root response to N for the improvement of N fertilizer application based on a scientific N management strategy.

## Materials and Methods

### Plant Materials and Culture Conditions

Experiments were carried out at the experimental station of Shandong Agricultural University, Tai’an, China (36°09′N, 117°09′E, 128 m above sea level). The Jimai 22 (JM22) winter wheat cultivar was grown under controlled conditions. Variations in temperature, illumination intensity, and relative humidity are shown in Supplementary Figure 1. Seeds were surface-sterilized with 1% NaClO_3_ for 30 min, rinsed 5 × with sterile water, and germinated on wet filter paper in the dark at 25°C for 2 days. Germinated seeds were sowed in plastic pots (10 cm × 10 cm × 10 cm; 10 plants/pot) filled with perlite. The pots were placed on plastic trays (55 cm × 45 cm × 5 cm; 20 pots/tray).

### Treatments and Experimental Design

Five NH_4_^+^/NO_3_^–^ ratio treatments with a 6-mM total N concentration were tested in a controlled climate chamber experiment. Treatments included: 100/0 (N_a_), 75/25 (N_r1_), 50/50 (N_r2_), 25/75 (N_r3_), and 0/100 (N_*n*_). Ca(NO_3_)_2_, NH_4_Cl, or NH_4_NO_3_ were used to set the ratios. The NH_4_^+^/NO_3_^–^ ratio preparation method is shown in Supplementary Table 1. The concentration of all other nutrient elements was referred to a modified Hoagland’s nutrient solution with the following chemical composition: 4 mmol L^–1^ Ca^2+^, 5 mmol L^–1^ KCl, 4 mmol L^–1^ MgSO_4_, 1 mmol L^–1^ KH_2_PO_4_, 50 μmol L^–1^ Fe-ethylenediaminetetraacetic acid (EDTA), 0.5 μmol L^–1^ H_3_BO_3_, 0.74 μmol L^–1^ MnSO_4_, 0.27 μmol L^–1^ ZnSO_4_, 0.001 μmol L^–1^ CuSO_4_, 0.001 μmol L^–1^ CoCl_2_, 0.005 μmol L^–1^ Na_2_MoO_4_, and 0.025 μmol L^–1^ KI; and pH 6.5. The nutrient solutions were supplemented once a day (1.5 L/tray). Wheat growth was evaluated from sowing (0 days) to 42 days after sowing (DAS). We observed that the first leaf emerged through the coleoptile at 7 DAS and unfolded at 14 DAS. The morphological characteristics of 42-day-old wheat plants under N_*n*_ treatment showed that four leaves were unfolded, and the fifth leaf was visible (Supplementary Figure 2). However, only three leaves were completely unfolded on 42-day-old wheat plants under N_*a*_ treatment.

### Measurements of the Shoot and Root Biomass Weight and Its Nitrogen Content Measurement

Ten wheat plants of each treatment were randomly sampled from 14 to 42 DAS at 7-day intervals. Sampled plants were divided into shoot and root. Samples were dried under 60°C for plant biomass weight measurement [g/plant dry weight (DW)].

The dried samples were pulverized, and their N content was measured using the semi-micro Kjeldahl method (Zou, 2000). The powder (0.1 g) was mixed with 3.2 g of the catalyzer (CuSO_4_/K_2_SO_4_, w/w: 1/15) and digested in 10 ml H_2_SO_4_ at 420°C for 2 h and cooled. Distillation was used as a FOSS Kjeltec 8200 analyzer (FOSS, Sweden). The boric acid is used as the receiving solution. The solution was titrated with sulfuric acid. The N content was calculated according to the following formula:


$$Nitrogencontent\left(\%\right)=\left(V_{\text{sample}}-V_{\text{blank}}\right)\times Nofacid\times 1.4÷W.$$

Where V_sample_ is the volume of used sulfuric acid for the sample (ml), V_blank_ is the volume of used sulfuric acid for blank (ml), N of acid represents normality of the used acid (mol L^–1^), and W is the sample dry weight (g).

### Measurements of Leaf Relative Chlorophyll Content and Chlorophyll Fluorescence Analysis

Eight intact fully expanded leaves of each treatment at 42 DAS were selected to measure relative chlorophyll content and the chlorophyll fluorescence analysis using the MultispeQ device (PhotosynQ, United States) according to the method described by Kuhlgert et al. (2016).

### Measurements of Root Growth

Root growth was evaluated by image analysis. Eight wheat plants of each treatment were sampled at 42 DAS. Roots were rinsed 3× with sterile water. Root sample images were captured with an EPSON V750 scanner (Seiko Epson Corp., Suwa, Nagano, Japan) at a resolution of 300 dpi. The images were analyzed in WinRHIZO (v. 4.0; Regent Instruments, Sainte-Foy, Quebec, Canada) which provided the total root length (TRL), root number (RN), and root average diameter (RAD). The root length and root biomass data were used to calculate the specific root length (SRL, TRL per unit of root dry weight, m g^–1^ DW).

### Root Nitrate and Ammonium Content Measurement

Roots of 10 wheat plants under each treatment were sampled from 14 to 42 DAS at 7-day intervals. The fresh root nitrate content was determined using the salicylic acid method, as described by Cataldo et al. (1975). Three replicates of 0.1 g wheat root from each treatment were grounded to a powder in liquid N_2_ and homogenized in 1 ml redistilled water. Samples were boiled for 30 min, cooled, and centrifuged at 12,000 × *g* for 15 min. For each sample, 0.1-ml supernatant was mixed with 0.4 ml salicylic acid–sulfate acid (5 g salicylic acid in 100 ml sulfate acid). The reactions were incubated at room temperature for 20 min, and 9.5 ml of 8% NaOH solution was added. After cooling the tube to room temperature, the optical density (OD)_410_ value was measured.

Ammonium concentrations in roots were measured using the O-phthalaldehyde (OPA) fluorometric method, as described by Schjoerring et al. (2002) and Wang et al. (2016b). Roots (0.5 g) were homogenized in liquid N_2_ and added 5 ml of 10 mM formic acid to extract NH_4_^+^. The root homogenates were centrifuged at 10,000 × *g* (4°C) for 15 min. Supernatants were transferred to 5 ml polypropylene tubes with 0.45-μm organic ultrafiltration membranes and centrifuged at 50,000 × *g* (4°C) for 10 min. A total of 100 ml of OPA reagent was prepared by combining 0.2 M potassium phosphate buffer (composed of equimolar amounts of potassium dihydrogen phosphate and potassium monohydrogen phosphate), 3.75 mM OPA, and 2 mM 2-mercaptoethanol, 1 day before use. Prior to adding 2-mercaptoethanol, the solution pH was adjusted to 7.0 using 1 M NaOH and filtered through two layers of filter paper. Extract (10 μl) was added to 3 ml of OPA reagent, and color was allowed to develop for 30 min in the absence of light (25°C) before sample absorbance was measured at 410 nm.

### Superoxide Anion Radical Measurement

Wheat roots under each treatment were sampled from 14 to 42 DAS at 7-day intervals. Anion radical (O_2_^–^) content and production rate were determined according to the method described by Bai et al. (2015). Three biological replicates of 0.5 g root samples for each treatment were homogenized in 3 ml of 65 mM phosphate buffer (pH 7.8), and the homogenate was centrifuged at 10,000 × *g* for 10 min. About 2 ml supernatant was added to 0.5 ml of 50 mM phosphate buffer (pH 7.8) and 0.1 ml of 10 mM hydroxylamine hydrochloride. After 20 min at 25°C, the mixture was added to 1 ml of 58 mM sulfanilamide and 1 ml of 7 mM α-naphthylamine, and let at 30°C for 30 min. After this period, the absorbance was measured at 530 nm.

### Root Lignin Content Measurement

Wheat roots of each treatment were sampled from 14 to 42 DAS at 7-day intervals. Root lignin contents were determined according to a modified method described by Zheng et al. (2017). Three replicates of 0.1 g root for each treatment were grounded to a powder in liquid N_2_ and washed five times with 80% ethanol to remove soluble metabolites, followed by acetone wash and dried in a drying oven. Samples were transferred to 10-ml centrifuge tubes with 2.5 ml acetyl bromide and acetic acid solution (v/v, 1:4) and incubated at 70°C for 1 h. After cooling to room temperature, 0.9 ml of 2 M NaOH was added to terminate the reaction, then mixed and added 0.1 ml of 7.5 M hydrochloride. Finally, 4 ml acetic acid was added to each tube. The solution absorbance was measured at 280 nm using a spectrophotometer. The lignin contents were calculated according to the following linear calibration curve:

Lignin content (mg g^–1^ FW) = (ΔA_280_ − 0.0068) ÷ 0.035 × V × 10^–3^ ÷ *W* × T.

Where ΔA_280_ is the absorbance at 280 nm, *W* is sample fresh weight in g, and T is the dilution factor. Calibration curves were generated with increasing concentrations of standard lignin (Sigma-Aldrich, Inc., St. Louis, MO, United States) (4, 8, 12, 16, 20, and 24 mg L^–1^), which were processed by the same method used for plant samples.

### Lignin Biosynthesis-Related Enzyme Activity Determination

Wheat roots of each treatment were sampled from 14 to 42 DAS at 7-day intervals. About 0.5 g root samples with three replicates from each treatment were ground to a powder in liquid N, and extracted with pH 7.0 phosphate buffer. The activities of PAL (EC:4.3.1.24), 4CL (EC:6.2.1.12), CCR (EC:1.2.1.44), CAD (EC:1.1.1.195), caffeic acid 3-O-methyltransferase (EC:2.1.1.68, COMT), and peroxidase (EC:1.11.1.7, POD) were measured using enzyme activity assay kits (Caobenyuan Biotechnology Co., Ltd., Nanjing, China).

### RNA Sequencing and Data Analysis

Roots were sampled in each treatment group at 42 DAS, immediately frozen in liquid N, and stored at −80°C until the RNA extraction and proteome analysis. Total RNA was extracted using the TRIzol reagent (Invitrogen, Carlsbad, CA, United States). Total RNA quantity and purity were determined using a NanoDrop and Agilent Bioanalyzer 2100 system (Agilent Technologies). Approximately 10 μg of total RNA representing a specific adipose type was subjected to isolation of poly (A) mRNA with poly T oligo-attached magnetic beads (Invitrogen, Carlsbad, CA, United States). Following purification, mRNA was fragmented into small pieces using divalent cations at high temperatures. Cleaved RNA fragments were reverse-transcribed to create the final cDNA library in accordance with the protocol by the manufacturer for the mRNA-Seq Sample Preparation Kit (Illumina, San Diego, CA, United States); the average insert size for the paired-end libraries was 300 bp (±50 bp). Finally, we performed paired-end sequencing on an Illumina HiSeq 4000 at the LC Sciences, San Diego, CA, United States, following the protocol recommended by the vendor.

The adaptor reads were removed from raw data using the Cutadapt software^1^. Clean data were obtained by removing the low-quality and repeat reads. Sequence quality was verified using the FastQC software^2^, including Q20 (phred quality score 20, percentage of bases whose base call accuracy exceeds 99%), Q30 (phred quality score 30, percentage of bases whose base call accuracy exceeds 99.9%), and guanine-cytosine (GC) content. We aligned the sample reads to the wheat reference genome of the International Wheat Genome Sequencing Consortium (IWGSC) RefSeq v1.0 (IWGSC 2018) using HISAT2. Mapped reads from each sample were assembled using the StringTie software. The value for fragment per kilobase of transcript per million mapped reads (FPKM) was calculated to quantify its expression abundance and variations using the StringTie software (version v1.3.3)^3^. DESeq2 software^4^ was used to analyze differentially expressed genes (DEGs) selected with the parameter false discovery rate (FDR) < 0.05, and an absolute fold change of ≥2. Gene ontology (GO) and the Kyoto Encyclopedia of Genes and Genomes (KEGG) databases were used to reveal the functional enrichment of DEGs.

### Protein Extraction and Identification

Root samples were ground under liquid N, and 0.8 g of the powder obtained was transferred to a 5-ml centrifuge tube, homogenized with 1 ml lysis buffer, and placed on ice for 10 min. Tris–phenol (1 ml) was added to the tube, homogenized, and placed again on ice for 10 min. Samples were then centrifuged at 16,000 × *g* for 10 min at 4°C. The supernatant was transferred into a new tube, and 4× volumes of cold ammonium acetate/methanol were added, followed by incubation at −20°C overnight. Then, samples were centrifuged at 16,000 × *g* for 10 min at 4°C, the supernatant was discarded, cold methanol was added, and the mixture was incubated at − 20°C for 1 h. After centrifugation at 16,000 × *g* for 10 min at 4°C, the sediment was collected, resuspended in cold acetone, washed two times, and air-dried. Samples were dissolved in 0.6 ml lysis buffer [8 M urea, 50 mM pH 8.0 Tris–HCl, 1% NP40, 1% sodium deoxycholate (NaDOC), 10 mM EDTA, 5 mM dithiothreitol (DTT), 1% protease inhibitor mixture, and 1% phosphatase inhibitor mixture], and centrifuged at 20,000 × *g* for 10 min at 4°C. The supernatant was collected, and the protein concentration was quantified using the Bio-Rad Protein Assay Kit (Bio-Rad, Hercules, CA, United States) following the instructions of the manufacturer. Then, 30 μg of protein from each sample was used to determine the quality of the proteins using 12% sodium dodecyl sulfate polyacrylamide gel electrophoresis (SDS-PAGE).

About 500 μg of protein from each sample was transferred to a new tube, adjusted to an equal volume with lysis buffer, and incubated at 30°C for 30 min. After cooling to room temperature, 30 mM iodoacetamide (IAM) was added, and the mixture was incubated at room temperature for 45 min in the dark, before adding 5× volumes of cold acetone, followed by incubation at −20°C overnight. After centrifugation at 20,000 × *g* for 10 min at 4°C, the sediment was collected, resuspended in cold 80% acetone, washed two times, and air-dried, followed by the addition of 300 μl of 0.1 M triethylammonium bicarbonate (TEAB) to the proteins and sonication on ice for 5 min. Trypsin (20 μg) was added to the samples and incubated at 37°C overnight. After incubation, 1% trifluoroacetic acid (TFA) was added to the mixture to terminate the reaction. The peptides were desalted using a C_18_ SPE column (Phenomenex, Torrance, CA, United States) and vacuum-dried prior to reconstitution in 0.5 M TEAB and processing according to the protocol of the manufacturer for tandem mass tag (TMT) kit (Thermo Fisher Scientific, Waltham, MA, United States). Tryptic peptides were fractionated by high-pH reverse-phase high-performance liquid chromatography (HPLC) using a Waters XBridge Shield C_18_ RP column (3.5 μm particles, 4.6 mm × 250 mm; Waters Corp., Milford, MA, United States). Peptides were separated into 60 fractions, combined into 20 fractions, and vacuum centrifugation-dried. The fractions were dissolved in 0.1% formic acid, centrifuged at 20,000 × *g* for 2 min at 4°C, and the supernatant was transferred to a sampler vial. Liquid chromatography with tandem mass spectrometry (LC–MS/MS) analysis was performed by Micrometer Biotech Company (Hangzhou, China).

Acquired MS/MS spectra were searched against the *Triticum aestivum* UniProt database (143,807 sequences). At least one unique peptide with an FDR of ≤1% was required for analysis of the protein identification and quantification data. For analysis of differentially expressed proteins (DEPs), only proteins with quantitative information from at least two biological replicates were used. The average of three biological replicates was used to indicate final protein abundance; finally, proteins showing an average protein-abundance change significantly greater than 1.3-fold (ANOVA test, *p* ≤ 0.05) were defined as DEPs.

### Statistical Analysis and Processing

Data for leaf chlorophyll content and chlorophyll fluorescence parameters, TRL, RAD, SRL, N content, O_2_^–^ content, lignin content, and lignin biosynthesis-related enzyme activity were processed in DPS v. 7.05 (Hangzhou, China). Multiple comparisons were performed after a preliminary *F*-test. The means were tested with a least significant difference test, and the significance was set at the probability level of 0.05. Graphs were plotted using OriginPro 2017 (OriginLab Inc., Northampton, MA, United States).

## Results

### Wheat Growth Responses to Increasing NH_4_^+^/NO_3_^–^ Ratios

**FIGURE 1:**
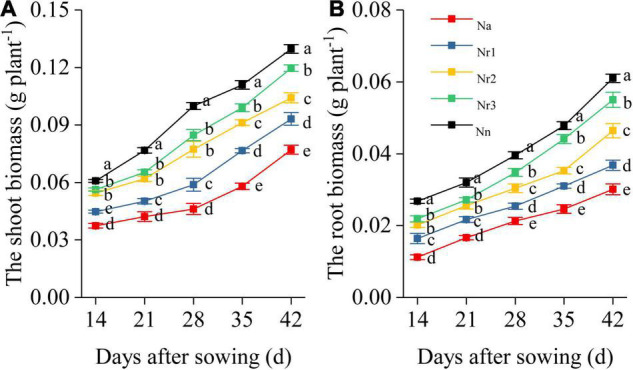
The effects of different NH_4_^+^/NO_3_^–^ ratios on the wheat shoot **(A)** and root biomass **(B)** from 14 to 42 days after sowing (DAS). Segments represent the SEM (*n* = 10). Different letters indicate significant differences among each treatment, *p* < 0.05.

Overall, NH_4_^+^/NO_3_^–^ ratio affected shoot and root biomass and morphological traits of wheat seedlings (Figure 1 and Supplementary Figure 2). Shoot and root biomass decreased with the increment of NH_4_^+^/NO_3_^–^ ratio. In particular, the shoot and root biomass exhibited significant differences among the five treatments at 35 and 42 DAS. For example, compared with the N_n_ treatment, shoot biomass in N_a_, N_r1_, N_r2_, and N_r3_ decreased by 40.56, 28.14, 19.66, and 7.79% at 42 DAS, respectively, whereas root biomass decreased by 50.66, 39.67, 23.93, and 9.84%, respectively (Figure 1).

**FIGURE 2:**
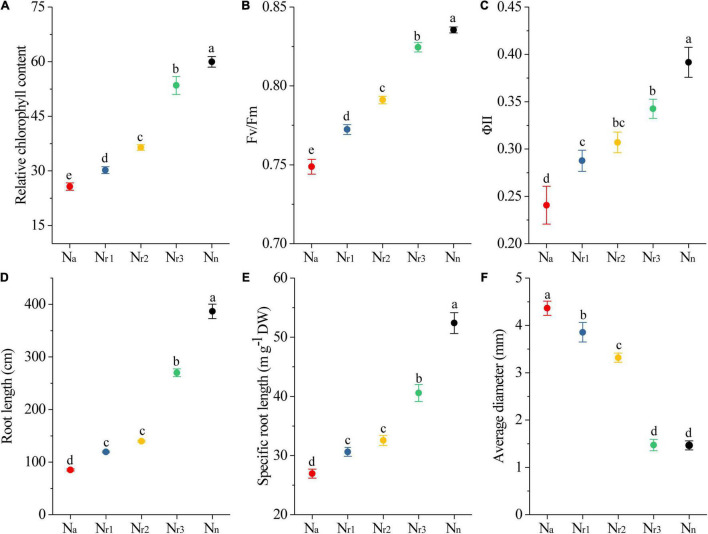
The effects of different NH_4_^+^/NO_3_^–^ ratios on leaf relative chlorophyll content **(A)**, Fv/Fm **(B)**, ΦII **(C)**, total root length **(D)**, specific root length **(E)**, and root average diameter **(F)**. Segments represent the SEM (*n* = 8). Different letters indicate significant differences among each treatment, *p* < 0.05.

As the NH_4_^+^/NO_3_^–^ ratio increased, the leaf relative chlorophyll content at 42 DAS decreased significantly (Figure 2A). In particular, the relative chlorophyll content decreased by 57.18% in the N_*a*_ treatment compared with the N_*n*_ treatment. In addition, the chlorophyll fluorescence analysis showed that both the maximal quantum efficiency of photosystem II (PSII) (Fv/Fm) and the steady-state efficiency (ΦII) decreased with increasing NH_4_^+^/NO_3_^–^ ratios (Figures 2B,C).

TRL and SRL decreased significantly as the NH_4_^+^/NO_3_^–^ ratio increased. Furthermore, the N_n_ treatment resulted in longer TRL and SRL but less RAD (Figures 2D–F). In contrast, the N_a_ treatment significantly (*p* < 0.05) reduced TRL and SRL by 77.98% and 48.59%, respectively, compared with the N_n_ treatment. Meanwhile, RAD increased with increasing NH_4_^+^/NO_3_^–^ ratios. Indeed, the RAD of the N_r1_ treatment increased by 280.21% compared to N_n_.

### Root Transcriptomic and Proteomic Responses to Increasing NH_4_^+^/NO_3_^–^ Ratios

Transcriptome sequencing generated from 3.80 to 5.50 million raw data. After quality control, over 97% of the reads with Q30 were saved for further analysis. On average, more than 70% of these reads were specific to wheat and uniquely aligned to the reference genome (Supplementary Table 2).

**FIGURE 3:**
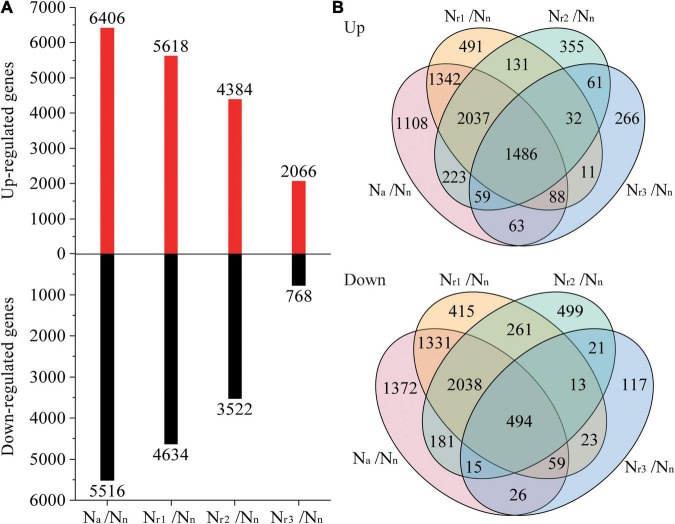
**(A)** Identification and comparison analysis of differentially expressed genes (DEGs) in the wheat root samples of different NH_4_^+^/NO_3_^–^ ratio treatments. **(B)** Venn diagrams represent up and downregulated genes in four comparisons.

**FIGURE 4:**
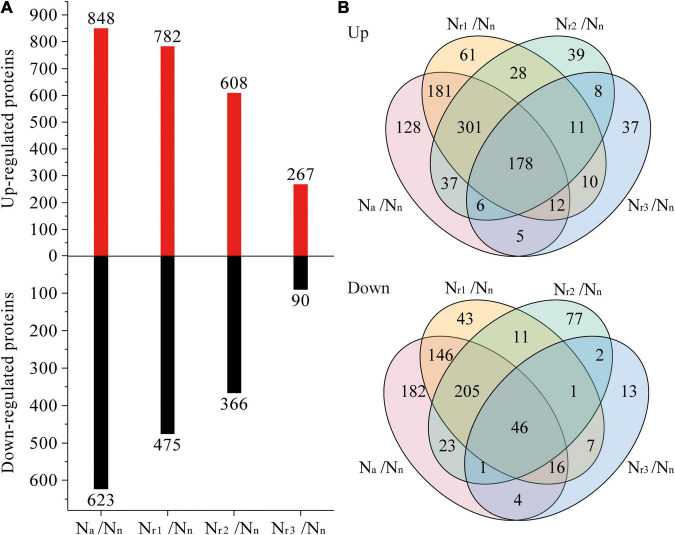
**(A)** Identification and comparison analysis of differentially expressed proteins (DEPs) in the wheat root samples of different NH_4_^+^/NO_3_^–^ ratio treatments. **(B)** Venn diagrams represent up and downregulated proteins in four comparisons.

A total of 7,650 (upregulated) and 6,726 (downregulated) DEGs were identified in the four pairwise comparisons listed in Supplementary Table 3. Concomitantly, a total of 1,042 (upregulated) and 777 (downregulated) DEPs were identified (Supplementary Table 4). Compared with the N_n_ treatment, increasing the NH_4_^+^/NO_3_^–^ ratio significantly increased the number of DEGs and DEPs (Figures 3A, 4A). A total of 2,834 DEGs (2,066 upregulated and 768 downregulated) and 357 DEPs (267 upregulated and 90 downregulated) were identified in the N_r3_/N_n_ group. Additionally, a total of 11,922 DEGs (6,406 upregulated and 5,516 downregulated) and 1,471 DEPs (848 upregulated and 623 downregulated) were detected in the N_a_/N_n_ group. The results of the Venn diagrams showed that 1,108 upregulated DEGs and 1,372 downregulated DEGs, and 128 upregulated DEPs and 182 downregulated DEPs were unique to N_a_/N_n_ (Figures 3B, 4B). Furthermore, a total of 266 upregulated DEGs and 117 downregulated DEGs, and 37 upregulated DEPs and 13 downregulated DEPs were unique to N_r3_/N_n_. The four groups shared 1,486 upregulated DEGs and 494 downregulated DEGs, and 178 upregulated DEPs and 46 downregulated DEPs.

### NH_4_^+^/NO_3_^–^ Ratio Changed Kyoto Encyclopedia of Genes and Genomes Enrichment Pattern of Differentially Expressed Genes and Differentially Expressed Proteins in Wheat Seedling Roots

Thirty-seven significant KEGG pathways were enriched for DEGs in the N_*a*_/N_n_ transcriptome comparison group (Supplementary Table 5), whereas 33 KEGG pathways were enriched in the N_r3_/N_*n*_ comparison group. Additionally, 24 pathways were shared, including glutathione metabolism (ko00480) and phenylpropanoid biosynthesis (ko00940) (Supplementary Figure 3A and Supplementary Table 5). Concomitantly, 13 significant KEGG pathways were enriched in the N_a_/N_n_ proteome comparison group, but only six pathways were enriched in the N_r3_/N_n_ group (Supplementary Table 6). Four pathways (ko00330, arginine, and proline metabolism; ko00620, pyruvate metabolism; ko00950, isoquinoline alkaloid biosynthesis; ko00960, tropane, piperidine, and pyridine alkaloid biosynthesis) were unique to N_a_/N_n_, and two pathways (ko00900, terpenoid backbone biosynthesis, and ko00780, biotin metabolism) were unique to N_r3_/N_n_ (Supplementary Figure 3B and Supplementary Table 6). Additionally, only glutathione metabolism (ko00480) and phenylpropanoid biosynthesis (ko00940) were shared among the four comparison groups.

### Glutathione Metabolism-Related Differentially Expressed Genes and Differentially Expressed Proteins Responses to Increasing NH_4_^+^/NO_3_^–^ Ratios

The four comparison groups identified a total of 236 DEGs and 53 DEPs related to the glutathione metabolism pathway (00480) (Supplementary Tables 7, 8). Thirty-one DEGs and nine DEPs were unique to N_*a*_/N_*n*_ (Supplementary Figure 4). Additionally, 82 DEGs and 12 DEPs were shared among the four comparison groups, all of which were upregulated, in which 81 DEGs were GSTs (EC:2.5.1.18) and only one gene (*TraesCS1A02G095800*) was an AP domain-containing protein (EC:3.4.11.1). Although only two proteins (A0A3B6K9P2 and Q8RW00) were GST, eight uncharacterized genes (A0A3B5XZ69, A0A3B6ER15, A0A3B6H0S6, A0A3B6I5M9, A0A3B6ILA6, A0A3B6JJE1, A0A3B6LUE0, and W5CW67) had GST molecular function, accordingly with the GO enrichment analysis. Finally, A0A3B6MRF8 may play a role in zinc-ion binding, whereas A0A3B6JL78 has POD activity.

### Phenylpropanoid Biosynthesis-Related Differentially Expressed Genes and Differentially Expressed Proteins Responses to Increasing NH_4_^+^/NO_3_^–^ Ratio

**FIGURE 5:**
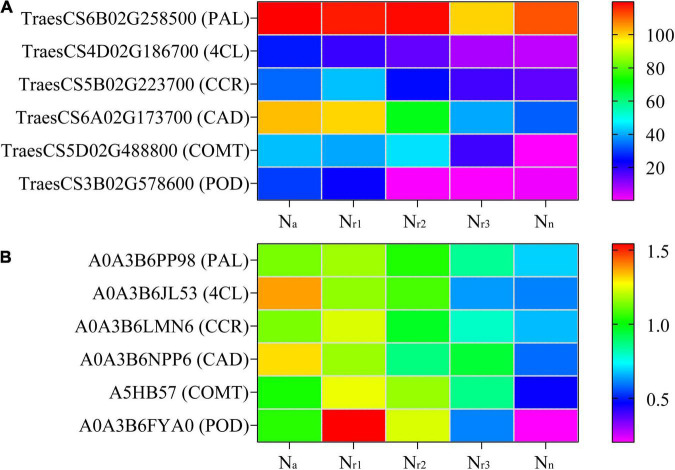
Heatmaps represent the candidate genes expression **(A)** and proteins abundance **(B)** involved in lignin biosynthesis in different NH_4_^+^/NO_3_^–^ ratios treatments. The gene expression was scaled using the fragment per kilobase of transcript per million mapped reads (FPKM) based on a mean value of three biological replicates. Different colors represent the upregulation and downregulation difference of genes and proteins among treatments. PAL, phenylalanine ammonia-lyase; 4CL, 4-coumarate-CoA ligase; CCR, cinnamoyl-CoA reductase; CAD, cinnamyl-alcohol dehydrogenase; COMT, caffeic acid 3-O-methyltransferase; POD, peroxidase.

A total of 598 DEGs and 70 DEPs related to the phenylpropanoid biosynthesis pathway (00940) were identified in the four comparison groups (Supplementary Tables 9, 10), and 99 DEGs and 12 DEPs were shared among the four comparison groups (Supplementary Figure 5). Based on the gene annotation, *TraesCS2D02G581200* was defined as 4CL (EC: 6.2.1.12). Seven upregulated genes were associated with shikimate O-hydroxycinnamoyl transferase (EC: 2.3.1.133, HCT). Fourteen upregulated genes and one downregulated gene were associated with CCR (EC: 1.2.1.44). One up and two downregulated genes were associated with COMT (EC: 2.1.1.68). Four upregulated and two downregulated genes were associated with CAD (EC: 1.1.1.195). Furthermore, 19 upregulated and 28 downregulated genes were associated with a POD (EC: 1.11.1.7). Based on the protein annotation, two PKS_ER domain-containing proteins (A0A3B6NPP6 and A0A3B6LM09) were obtained, whose amino acid sequences were aligned to those of the corresponding proteins in the wheat genome. Results of the phylogenetic tree constructed showed that both A0A3B6NPP6 and A0A3B6LM09 belong to the CAD subfamily (Supplementary Figure 6). Six upregulated and three downregulated proteins were associated with POD. Related genes encoding phenylalanine ammonia-lyase (PAL), 4CL, CCR, CAD, COMT, and POD were upregulated as NH_4_^+^/NO_3_^–^ ratio increase, along with the related proteins (Figure 5).

### Nitrogen Metabolism and Transport-Related Differentially Expressed Genes and Differentially Expressed Proteins in Response to Increasing NH_4_^+^/NO_3_^–^ Ratios

**FIGURE 6:**
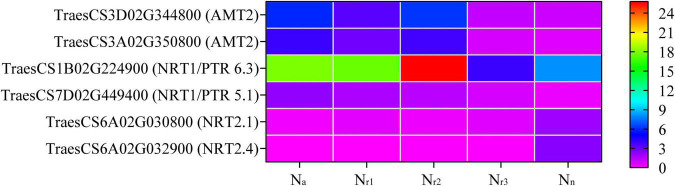
Heatmaps represent the candidate genes expression involved in nitrogen transport in different NH_4_^+^/NO_3_^–^ ratios treatments. The gene expression was scaled using the FPKM based on a mean value of three biological replicates. Different colors represent the upregulation and downregulation difference of genes and proteins among treatments. AMT2, ammonium transporter 2; NRT1/PTR6.3, nitrate transporter 1/peptide transporter family 6.3; NRT1/PTR5.1, nitrate transporter 1/peptide transporter family 5.1; NRT2.1, high-affinity nitrate transporter 2.1; NRT2.4, high-affinity nitrate transporter 2.4.

A total of 68 DEGs and 12 DEPs involved in the N metabolism pathway (00910) were identified in all four comparison groups (Supplementary Tables 11, 12). Seven DEGs and three DEPs were unique to N_a_/N_n_, and one DEG and one DEP were unique to N_r1_/N_n_ (Supplementary Figure 7). Additionally, the four comparison groups shared 27 DEGs and three DEPs. Based on the gene annotation and description, two upregulated DEGs (*TraesCS3D02G344800* and *TraesCS3A02G350800*) were involved in ammonium transport (*AMT*) in the N_a_/N_n_, N_r1_/N_n_, and N_r2_/N_n_ comparison groups (Supplementary Table 13). In the four comparison groups, 15 downregulated DEGs were high-affinity nitrate transporters or nitrate transporters, including *NRT2.1* and *NRT2.4*. Furthermore, the upregulated *TraesCS7D02G449400* in the four comparison groups belongs to the *NRT1/PTR Family 5.1*. *TraesCS1B02G224900* (*NRT1/PTR Family 6.3*) was downregulated in the N_r3_/N_n_ group but upregulated in the N_a_/N_n_, N_r1_/N_n_, and N_r2_/N_n_ comparison groups (Figure 6 and Supplementary Table 13). Five DEGs were involved in nitrate reduction, and seven showed carbonic anhydrase activity. Based on protein annotation, the downregulated *A0A3B6Q9B3* gene was involved in the nitrate transport in the four comparison groups and was successfully mapped to *TraesCS6D02G035600*. A0A3B6PQS3 was involved in the ferredoxin-nitrite reduction and mapped to *TraesCS6B02G364600*. A0A3B6TX28 showed to have carbonic anhydrase activity and was mapped to *TraesCS7D02G443400*.

### Effect of Increasing NH_4_^+^/NO_3_^–^ Ratios on Nitrogen, NO_3_^–^-N, and NH_4_^+^-N Contents in Wheat Seedlings

**FIGURE 7:**
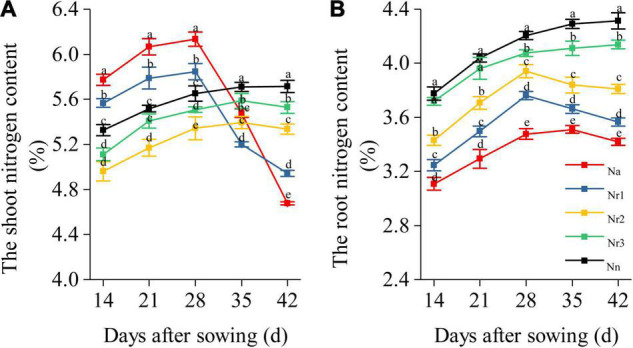
The effects of different NH_4_^+^/NO_3_^–^ ratios on the nitrogen content of the shoot and **(A)** root **(B)** from 14 to 42 days after sowing (DAS). Segments represent the SEM (mean ± SE, *n* = 3). Different letters indicate significant differences among each treatment, *p* < 0.05.

The result showed that the shoot N content of N_a_ and N_r1_ treatments gradually increased but sharply decreased from 35 to 42 DAS (Figure 7A). Nevertheless, the shoot N content of N_n_, N_r2_, and N_*r*3_ treatments increased from 14 to 42 DAS. Finally, the shoot N content decreased significantly with increasing NH_4_^+^/NO_3_^–^ ratios at 42 DAS. For example, the shoot N content decreased by 6.64 and 13.52% after N_r2_ and N_r1_ treatments compared with N_n_, respectively. Similarly, the root N content of N_n_ and N_r3_ treatments increased from 14 to 42 DAS (Figure 7B), whereas the root N content of N_a_, N_r1_, and N_r2_ treatments gradually increased, and they later decreased from 35 to 42 DAS. The root N content significantly decreased with increasing NH_4_^+^/NO_3_^–^ ratios. Relative to the N_r3_ treatment, the root N content decreased by 13.83 and 17.37% at 42 DAS in N_r1_ and N_a_ treatments, respectively.

**FIGURE 8:**
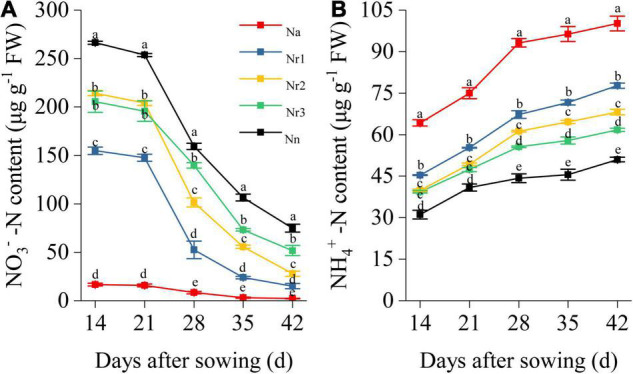
The effects of different NH_4_^+^/NO_3_^–^ ratios on root NO_3_^–^-N **(A)** and NH_4_^+^-N content **(B)** from 14 to 42 days after sowing (DAS). Segments represent the SEM (mean ± SE, *n* = 3). Different letters indicate significant differences among each treatment, *p* < 0.05.

The root NO_3_^–^-N content gradually decreased with plant growth. Conversely, the NH_4_^+^-N content gradually increased with plant growth (Figure 8). Increasing NH_4_^+^/NO_3_^–^ ratios significantly reduced the NO_3_^–^-N content in wheat seedling roots (Figure 8A). Thus, the N_a_ treatment yielded the lowest root NO_3_^–^-N content, whereas the N_n_ treatment showed the highest NO_3_^–^-N content. Indeed, at 42 DAS, the NO_3_^–^-N content was 71.07% lower in the N_r1_ treatment than in the N_r3_ treatment. Increasing the NH_4_^+^/NO_3_^–^ ratios significantly increased the NH_4_^+^-N content in roots. The highest NH_4_^+^-N content was observed for the N_a_ treatment, which was 96.35% higher than that of the N_n_ treatment at 42 DAS (Figure 8B).

### Effects of the NH_4_^+^/NO_3_^–^ Ratio on O_2_^–^ Generation Rate and Content in Wheat Seedling Roots

**FIGURE 9:**
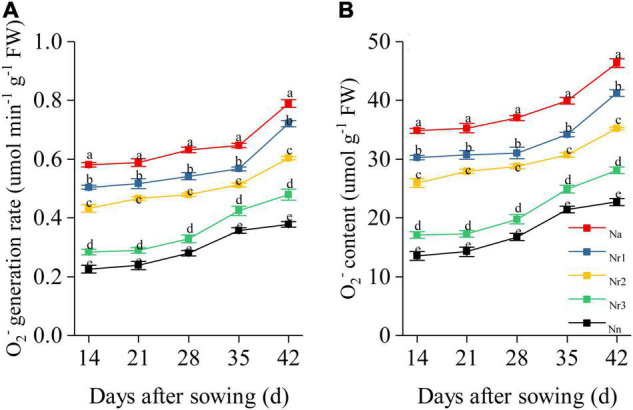
The effects of different NH_4_^+^/NO_3_^–^ ratios on root O_2_^–^ generation rate **(A)** and O_2_^–^ content **(B)** from 14 to 42 days after sowing (DAS). Segments represent the SEM (mean ± SE, *n* = 3). Different letters indicate significant differences among each treatment, *p* < 0.05.

Increasing the NH_4_^+^/NO_3_^–^ ratio significantly promoted O_2_^–^ generation and increased O_2_^–^ content in wheat seedling roots (Figure 9). For example, the N_a_ treatment yielded the highest O_2_^–^ generation rate and content in the roots. Thus, compared with the N_n_ treatment, O_2_^–^ content was enhanced by 104.20% in the N_*a*_ treatment at 42 DAS; consistently, compared with the N_r3_ treatment, the O_2_^–^ generation rate was 50.41% higher under the N_r3_ treatment at 42 DAS. Relative to the N_r3_ treatment, the O_2_^–^ content in the N_r1_ treatment increased by 46.85% at 42 DAS.

### Effects of the NH_4_^+^/NO_3_^–^ Ratio on Lignin Content and the Activity of Lignin Biosynthesis-Related Enzymes in Wheat Seedling Roots

**FIGURE 10:**
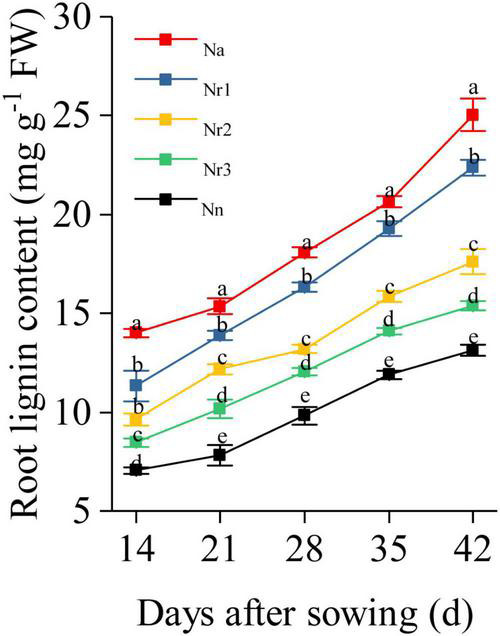
The effects of different NH_4_^+^/NO_3_^–^ ratios on root lignin content from 14 to 42 days after sowing (DAS). Segments represent the SEM (mean ± SE, *n* = 3). Different letters indicate significant differences among each treatment, *p* < 0.05.

**FIGURE 11:**
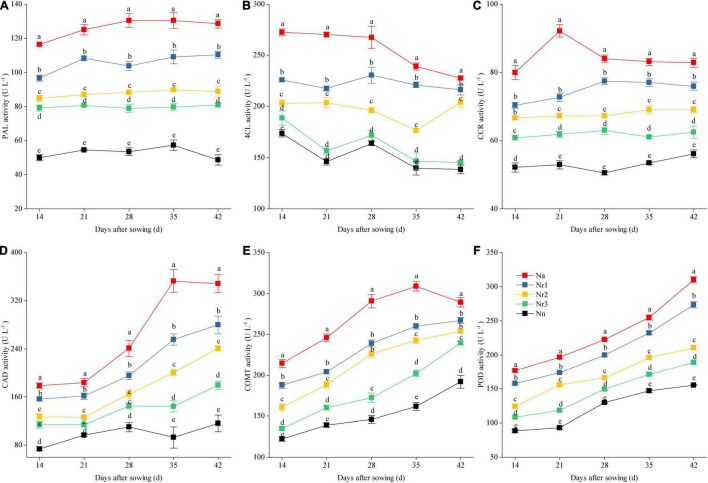
The effects of different NH_4_^+^/NO_3_^–^ ratios on root PAL **(A)**, 4CL **(B)**, CCR **(C)**, CAD **(D)**, COMT **(E)**, and POD **(F)** activities from 14 to 42 days after sowing (DAS). Segments represent the SEM (mean ± SE, *n* = 3). Different letters indicate significant differences among each treatment, *p* < 0.05. PAL, phenylalanine ammonia-lyase; 4CL, 4-coumarate-CoA ligase; CCR, cinnamoyl-CoA reductase; CAD, cinnamyl-alcohol dehydrogenase; COMT, caffeic acid 3-O-methyltransferase; POD, peroxidase.

The root lignin content increased gradually with increasing growth (Figure 10); furthermore, it increased significantly with increasing NH_4_^+^/NO_3_^–^ ratio. For instance, the lignin level for the N_a_ treatment was 90.80% higher than that for the N_n_ treatment at 42 DAS. Similarly, compared with the N_r3_ treatment, the lignin content was 45.46% higher in the N_r1_ treatment at 42 DAS. The observed increased lignin contents may be due to the altered activities of lignin biosynthesis-related enzymes. Similarly, an increasing NH_4_^+^/NO_3_^–^ ratio significantly increased PAL, 4CL, CCR, CAD, COMT, and POD activities (Figure 11). For instance, at 42 DAS, root PAL, 4CL, CCR, CAD, COMT, and POD activities in the N_r1_ treatment increased by 36.43, 48.72, 21.55, 56.24, 11.57, and 44.79%, respectively, relative to the N_r3_ treatment.

## Discussion

### Effects of Increasing NH_4_^+^/NO_3_^–^ Ratios on Wheat Seedling Growth

Nitrogen is an essential nutrient for plant growth, and consequently, the absorption of soil N is the basis for dry matter formation and accumulation. NH_4_^+^ and NO_3_^–^ are the two main N forms plants can absorb and utilize (Miller and Cramer, 2005). As new roots and leaves emerge during growth, wheat seedlings become increasingly sensitive to N availability (Yang et al., 2017). In previous studies, the high NH_4_^+^-N concentration could produce apparent stress, inhibiting plant growth and reducing biomass (Wang et al., 2016b; Vega-Mas et al., 2019). However, the wheat growth response to different NH_4_^+^/NO_3_^–^ ratios have different assessments. For example, Ijato et al. (2021) reported that ammonium-nitrate (NH_4_^+^/NO_3_^–^ ratio: 1/1) was better for wheat growth. Conversely, the results of our study were not in line with this finding. In this study, we conducted experiments with different NH_4_^+^/NO_3_^–^ ratio treatments to investigate the effects of the NH_4_^+^/NO_3_^–^ ratio on wheat seedling growth. We found that the NO_3_^–^-N treatment resulted in the highest wheat growth. As the NH_4_^+^/NO_3_^–^ ratio increased, significant growth inhibition occurred (Supplementary Figure 2). Although the sensitivity of different wheat cultivars to ammonium stress has been reported, wheat has been described as a species that is particularly sensitive to high NH_4_^+^ concentrations (Setién et al., 2013; Wang et al., 2016b; Liu et al., 2017). From this aspect, we used a wheat variety that is sensitive to the presence of ammonium. Indeed, wheat plants exhibited severe symptoms of ammonium toxicity. Moreover, the leaf chlorophyll content was significantly decreased with the increment in the NH_4_^+^/NO_3_^–^ ratio (Figure 2A). This may be due to the activation of a series of genes by NH_4_^+^, including *AMOS1*/*EGY1*, which participated in the regulation of NH_4_^+^-stress signaling (Li et al., 2012; Li B. et al., 2014). High NH_4_^+^ damaged chloroplast ultrastructure and decreased the abundance of proteins and transcripts in chloroplasts, such as *PsaA* (Shi et al., 2020; Zhou et al., 2021). The chlorophyll content is a proxy of the leaf photosynthetic capacity (Croft et al., 2017), and our results showed that the lower chlorophyll content was unfavorable by the photosynthate accumulation. Furthermore, the chlorophyll fluorescence analysis indicated that leaf Fv/Fm and ΦII were significantly reduced by an increasing NH_4_^+^ supply (Figures 2B,C). Chlorophyll fluorescence traits have been used to evaluate the effects of abiotic stress factors on photosynthesis (Sharma et al., 2017). Thus, Fv/Fm reflects the maximum quantum efficiency of the photochemical reactions mediated by PSII (Sharma et al., 2015). In turn, ΦII correlates well with net photosynthetic rate and effective quantum yield of CO_2_ fixation (Krall and Edwards, 1992). Ammonium directly accelerates the photodamage of PSII and affects the repair of photodamaged PSII (Dai et al., 2014). Ammonium also stimulates electron transport to photorespiration to detriment of Rubisco carboxylation (Alencar et al., 2019). In this study, Fv/Fm and ΦII decrease, indicating that a high NH_4_^+^/NO_3_^–^ ratio induces severe damage to the photosynthetic apparatus.

On the other hand, root growth was influenced by NH_4_^+^/NO_3_^–^ ratio. Our study showed that a high NH_4_^+^/NO_3_^–^ ratio changed root biomass and morphology (Figure 1B and Supplementary Figure 2). Furthermore, TRL and SRL were significantly reduced by increasing NH_4_^+^/NO_3_^–^ ratios despite an increase in RAD (Figures 2D–F). Root architecture plays an essential role in N uptake (Meister et al., 2014). SRL and root diameter are considered useful root traits to evaluate nutrient absorption (Li H. et al., 2014; Sharma et al., 2021). Moreover, SRL positively correlated with the total amount of N uptake; in contrast, average root diameter negatively correlated with N uptake capacity (Hong et al., 2018). These results showed that increasing NH_4_^+^/NO_3_^–^ ratios resulted in lower leaf photochemical efficiency and root uptake capacity, thereby reducing shoot and biomass and leading to stunted growth (Figure 1A and Supplementary Figure 2).

### Increasing NH_4_^+^/NO_3_^–^ Ratios Enhanced Root Glutathione Metabolism and Lignification, Thus Increasing Root Tolerance to Oxidative Damage but Reducing Nitrogen Transport and Utilization

In this study, we combined transcriptome with TMT-based proteome profiling to detect DEGs and DEPs and used functional predictions to explore enriched pathways that contribute to the root development under increasing NH_4_^+^/NO_3_^–^ ratio conditions. A total of 14,376 DEGs and 1,819 DEPs were identified across the comparisons (Supplementary Tables 3, 4). Multiple approaches have shown that high ammonium concentrations are strongly phytotoxic and promote ROS formation, leading to oxidative stress (Bittsánszky et al., 2015; Podgórska and Szal, 2015). Increasing NH_4_^+^ supply may play a role in the oxidative stress by enhancing transcripts level and the NADPH oxidase activity, which is known as respiratory-burst oxidase homologs (RBOHs), which can oxidize cytosolic NADPH and transfer an electron across the cell membrane to generate O_2_^–^ in the cytosol and cell wall (Swanson and Gilroy, 2010; Podgórska et al., 2021). In our study, we found that two upregulated DEGs were involved in the respiratory-burst oxidase expression (Supplementary Table 3). *TraesCS1B02G295300* was upregulated in all four comparison groups. Compared with N_n_, *TraesCS1B02G295200* was upregulated in the N_a_, N_*r*1_, and N_r2_ treatments. This explains why increasing the NH_4_^+^/NO_3_^–^ ratios significantly accelerated O_2_^–^ generation and increased O_2_^–^ content in the wheat roots (Figure 9). ROS act as signaling molecules regulating lateral root emergence and primary root elongation (Tsukagoshi, 2016; Liu and von Wirén, 2017). However, oxidative stress occurs when the enhanced production of ROS exceeds their degradation (Choudhary et al., 2020), thereby triggering several defense mechanisms in plants. Our KEGG pathway enrichment analysis showed that glutathione metabolism (ko00480) and phenylpropanoid biosynthesis (ko00940) were the main two shared pathways across comparisons (Supplementary Tables 5, 6). A total of 236 DEGs and 53 DEPs were involved in glutathione metabolism (Supplementary Tables 7, 8). Previous studies have found that GSTs are ubiquitous and multifunctional enzymes encoded by large gene families and have diverse roles in plant development and stress tolerance (Nianiou-Obeidat et al., 2017; Gullner et al., 2018). In our study, 81 upregulated genes were shared across comparisons and encoded GSTs involved in glutathione metabolism. According to the results of our proteome analysis, two shared upregulated proteins (A0A3B6K9P2 and Q8RW00) were GSTs. Previous studies reported that GSTs regulate root development by participating in auxin binding or transport (Jiang et al., 2010; Nongmaithem et al., 2021). GSTs also possess glutathione peroxidase activity, quench ROS with the addition of glutathione, and protect the cell from oxidative damage (Halušková et al., 2009; Kumar and Trivedi, 2018). We found that A0A3B6JL78 was described as a POD induced by a high NH_4_^+^/NO_3_^–^ ratio. Thus, the increased GST gene and protein expression under a high NH_4_^+^/NO_3_^–^ ratio may contribute to regulating root growth and avoiding O_2_^–^ accumulation.

Recent reports have shown that root lignification is another defense mechanism that plays a critical role in adapting to oxidative stress (Cesarino, 2019; Yan et al., 2019). Higher root lignification contributes to increasing ammonium tolerance in the cell (Royo et al., 2019). In this study, we found that a high NH_4_^+^/NO_3_^–^ ratio significantly increased root lignin content (Figure 10), likely because ROS directly modify the structure of the root cell wall by their involvement in lignin formation (Tsukagoshi, 2016). Indeed, ROS facilitates POD activity in the presence of NH_4_^+^ (Podgórska et al., 2021). Furthermore, we found that increasing the NH_4_^+^/NO_3_^–^ ratios significantly increased the activities of PAL, 4CL, CCR, CAD, COMT, and POD (Figure 11), which are key enzymes involved in lignin biosynthesis. Moreover, the transcriptome and proteome analyses showed 598 DEGs and 70 DEPs related to phenylpropanoid biosynthesis (Supplementary Tables 9, 10). DEGs and DEPs related to PAL, 4CL, CCR, CAD, COMT, and POD were upregulated as the NH_4_^+^/NO_3_^–^ ratio increased (Figure 5). In particular, we found two proteins belonging to the CAD subfamily, A0A3B6NPP6 and A0A3B6LM09, which were detected in the DEP data through phylogenetic comparison of the members of CAD among wheat genotypes (Supplementary Figure 6). Six upregulated proteins associated with POD were identified in each NH_4_^+^/NO_3_^–^ ratio treatment. These results indicated that increasing the NH_4_^+^/NO_3_^–^ ratios increased the expression of genes and proteins related to lignin biosynthesis, which contributed to reinforcing the root cell wall.

Excess lignin deposition in roots reduces the diameter of xylem vessels and restricts xylem extensibility, thereby limiting nutrient transport (Khandal et al., 2020). The increment in lignin deposition in the cell wall induces the formation of a root barrier that effectively reduces solute permeability and leads to reducing N transport in the roots (Ranathunge et al., 2016; Plett et al., 2020). Our results showed that increasing root NH_4_^+^/NO_3_^–^ ratios increased NH_4_^+^ but reduced NO_3_^–^ content in roots (Figure 8). According to the results of the transcriptome analysis, *TraesCS3D02G344800* and *TraesCS3A02G350800* (ammonium transporter) were upregulated under the high NH_4_^+^ conditions (N_*a*_, N_*r*1_, and N_*r*2_ treatments) (Figure 6). This could explain why the root had more NH_4_^+^-N content with the NH_4_^+^/NO_3_^–^ ratio increment. *NRT2* is a high-affinity transporter, especially under low N availability conditions (Tong et al., 2020). However, a previous study reported that *TaNRT2.1* could be expressed under 5 and 10 mmol^–1^ N (Wang et al., 2011). A recent study also reported that *Ta6A* (*TraesCS6A02G030800*) was at or near saturation at 0.5 mM N, but it was still taking up nitrate at 10 mM nitrate (Li et al., 2021). In our study, 15 shared downregulated DEGs were identified in the N metabolism pathway, which were involved in high-affinity nitrate transport or nitrate transport, including *NRT2.1* and *NRT2.4* (Figure 6 and Supplementary Tables 11, 12). This explained reduced root NO_3_^–^-N content with increasing NH_4_^+^/NO_3_^–^ ratios. Furthermore, a total of 12 DEPs involved in N metabolism were identified, among which A0A3B6Q9B3 was a nitrate transporter, and A0A3B6PQS3 was a ferredoxin-nitrite reductase. As a result, root N accumulation decreased significantly with increasing NH_4_^+^/NO_3_^–^ ratios (Figure 7B). Correlation analysis indicated that root lignin accumulation negatively correlated with NO_3_^–^ content but positively correlated with NH_4_^+^ content (Supplementary Figure 8). Based on the above, the increase in lignin deposition may decrease the N absorption ability of roots. These results demonstrate that increasing the NH_4_^+^/NO_3_^–^ ratios in roots of wheat seedlings were unfavorable for root N uptake and transport due to excessive root lignification.

## Conclusion

In the present study, the increment in the NH_4_^+^/NO_3_^–^ ratios reduced leaf chlorophyll content, Fv/Fm, and ΦII values in the ammonium sensitive wheat, indicating that a high NH_4_^+^/NO_3_^–^ ratio reduces leaf photosynthetic capacity. In addition, increasing the NH_4_^+^/NO_3_^–^ ratios caused O_2_^–^ generation in the roots and increased the activity of enzymes involved in lignin biosynthesis, leading to root growth inhibition. Under a high NH_4_^+^/NO_3_^–^ ratio, we identified upregulated DEGs and DEPs related to glutathione metabolism and phenylpropanoid biosynthesis in roots, which may contribute to an increase of root tolerance to oxidative damage. Furthermore, increasing the NH_4_^+^/NO_3_^–^ ratios downregulated DEGs and DEPs related to N metabolism and nitrate transport, leading to a decrease in root N content. These results show that high NH_4_^+^/NO_3_^–^ ratios in young wheat seedlings were unfavorable for root N uptake and transport due to excess lignification of the root cell wall.

## Data Availability Statement

The datasets presented in this study can be found in online repositories. The names of the repository/repositories and accession number(s) can be found below: https://figshare.com/articles/dataset/_/16826116.

## Author Contributions

DY conceived and designed the experiments, analyzed the data, prepared figures and tables, authored and reviewed drafts of the manuscript, and approved the final draft. JZ, CB, and LL performed the experiments. ZW contributed reagents, materials, analysis tools, and authored and reviewed drafts of the manuscript. All the authors contributed to the article and approved the submitted version.

## Conflict of Interest

The authors declare that the research was conducted in the absence of any commercial or financial relationships that could be construed as a potential conflict of interest.

## Publisher’s Note

All claims expressed in this article are solely those of the authors and do not necessarily represent those of their affiliated organizations, or those of the publisher, the editors and the reviewers. Any product that may be evaluated in this article, or claim that may be made by its manufacturer, is not guaranteed or endorsed by the publisher.

## Footnotes

1
https://cutadapt.readthedocs.io/en/stable/

2
http://www.bioinformatics.babraham.ac.uk/projects/fastqc/

3
http://ccb.jhu.edu/software/stringtie/

4
https://bioc.ism.ac.jp/packages/3.0/bioc/html/DESeq2.html
