# Supplementary material for: Transcriptome and Proteomics Analysis of Wheat Seedling Roots Reveals That Increasing NH4+/NO3– Ratio Induced Root Lignification and Reduced Nitrogen Utilization
Source: Front Plant Sci. 2022 Jan 13;12:797260. doi: 10.3389/fpls.2021.797260 (PMC8792948; doi:10.3389/fpls.2021.797260)
Supplement: Supplementary file 1 [file Data_Sheet_1.DOCX]

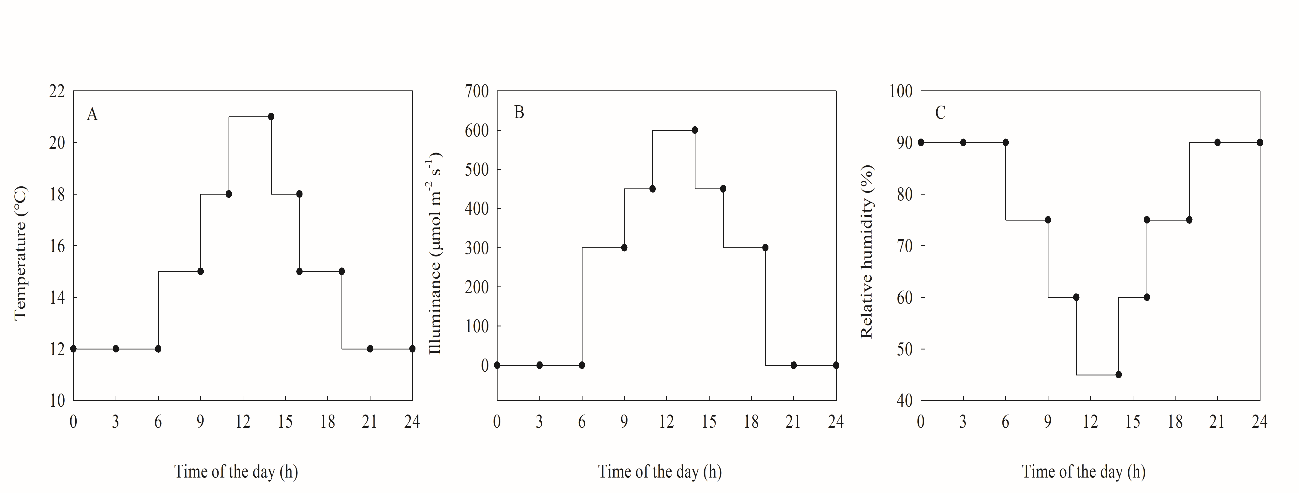


**Fig.S1 Daily changes of temperature (A), illumination intensity (B), and relative humidity (C) in the plant growth chamber.**


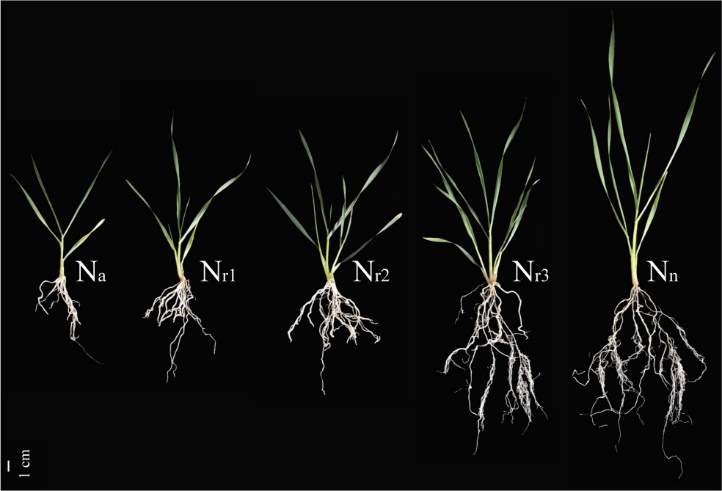


**Fig.S2 The effects of different NH_4_^+^/NO_3_^-^ ratios on wheat plant** **morphology.**


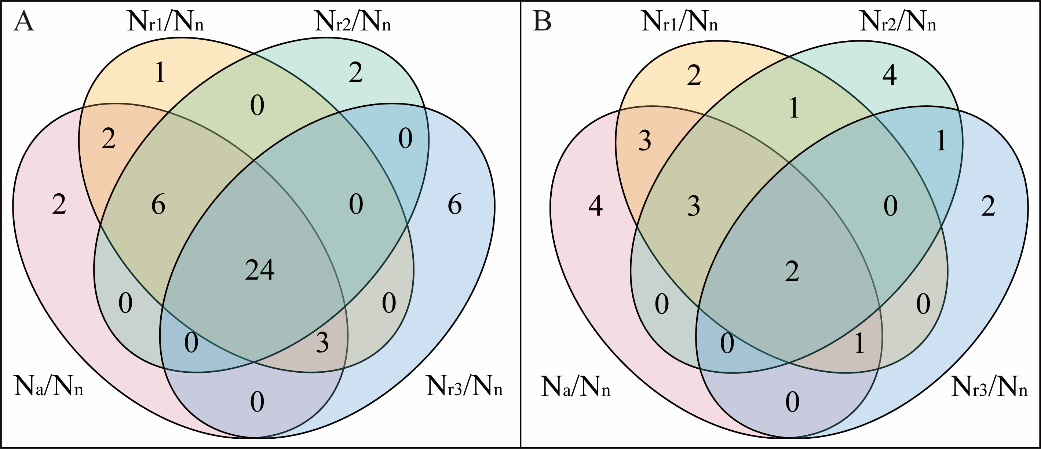


**Fig.S3** **Venn diagrams of enriched gene KEGG pathways (A) and protein KEGG pathways (B) in four comparisons.**


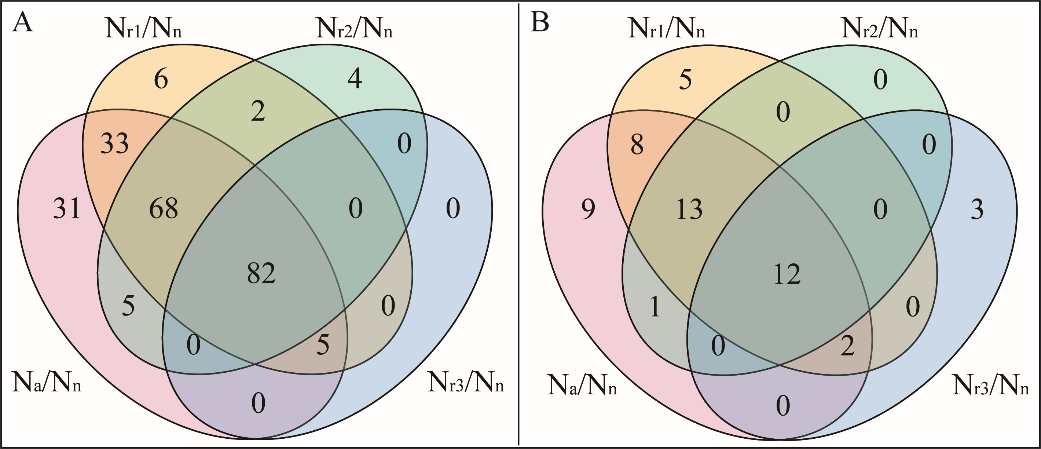


**Fig.S4 Venn diagrams of DEGs (A) and DEPs (B) involved in glutathione metabolism in four comparisons.**


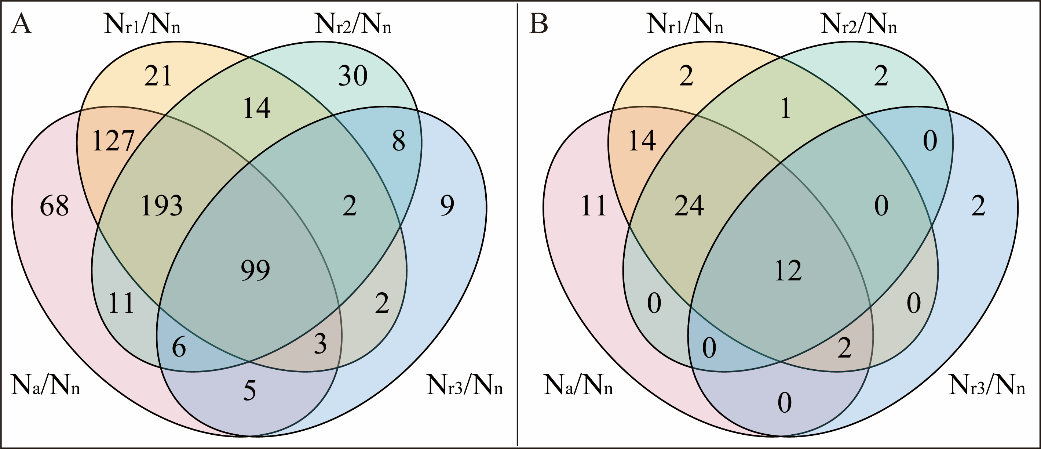


**Fig.S5 Venn diagrams of DEGs (A) and DEPs (B) involved in phenylpropanoid biosynthesis in four comparisons.**


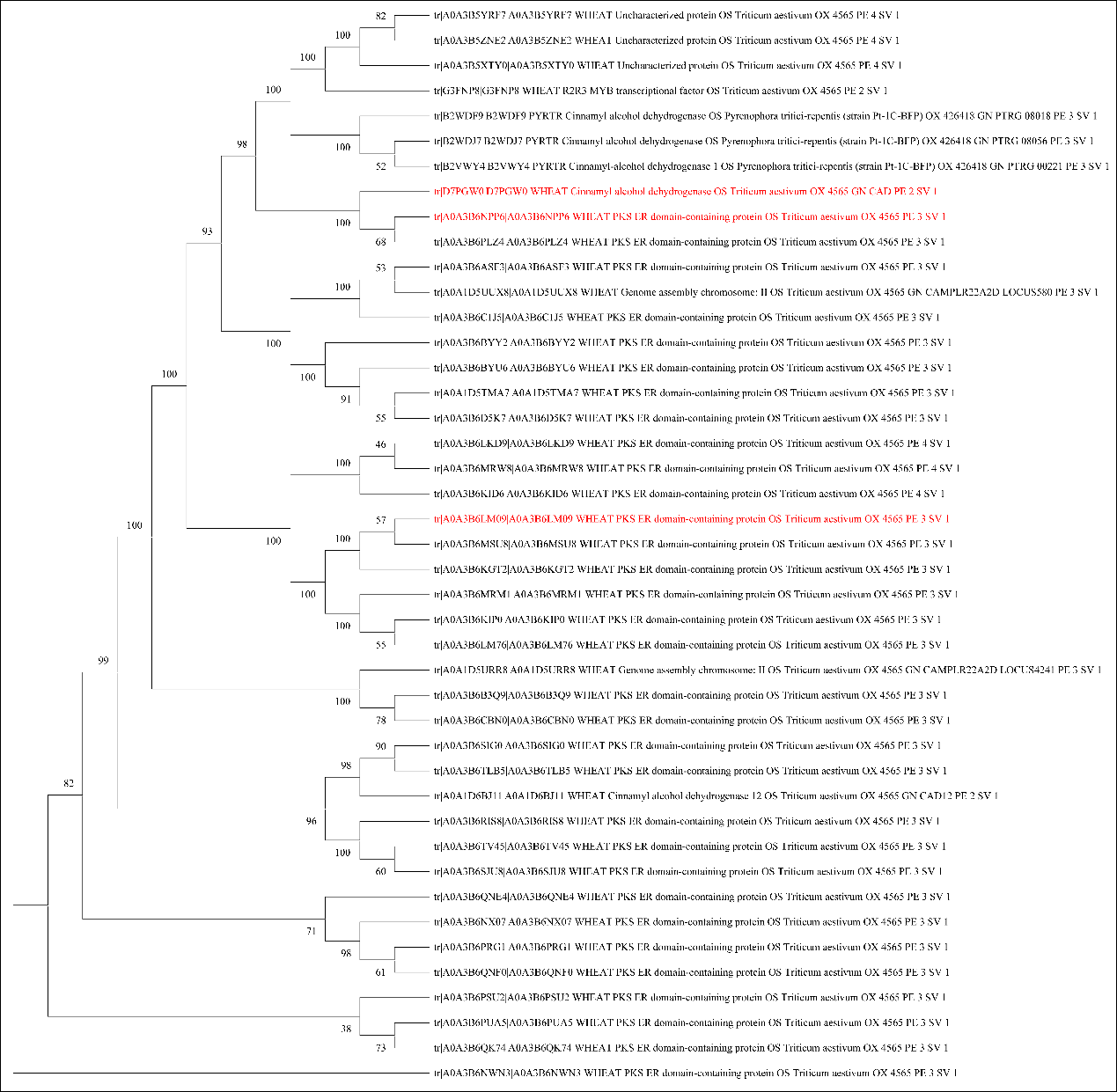


**Fig.S6 Phylogenetic relationships of *A0A3B6NPP6*, *A0A3B6LM09* and wheat CAD proteins.** The complete amino acid sequences of the 43 wheat CAD proteins were download from the UniProt database, and the Neighbor–Joining tree was constructed using MEGA.


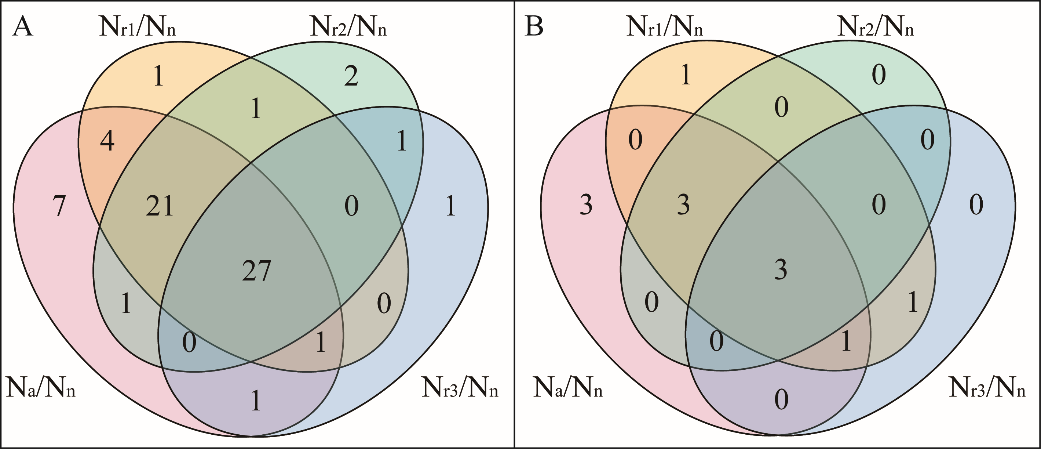


**Fig.S7 Venn diagrams of DEGs (A) and DEPs (B) involved in nitrogen metabolism in four comparisons.**


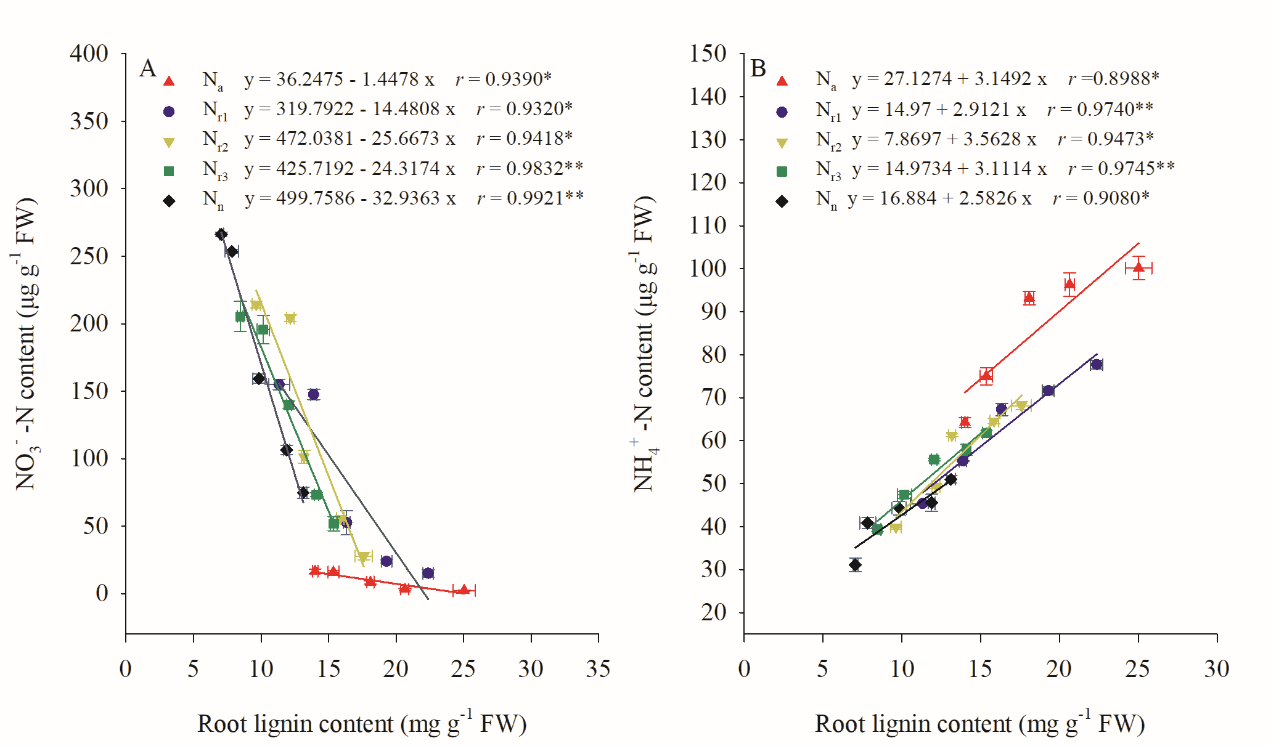


**Fig.8 The relationship between root lignin content and NO_3_^-^ -N (A), and NH_4_^+^ -N (B) content in each treatment.** Regression functions and coefficients of determination are given in each panel. * Significant correlation at P < 0.05. ** Significant correlation at P < 0.01.
