# Supplementary material for: Transcriptome and Proteomics Analysis of Wheat Seedling Roots Reveals That Increasing NH4+/NO3– Ratio Induced Root Lignification and Reduced Nitrogen Utilization
Source: Front Plant Sci. 2022 Jan 13;12:797260. doi: 10.3389/fpls.2021.797260 (PMC8792948; doi:10.3389/fpls.2021.797260)
Supplement: Supplementary file 2 [file Table_1.docx]

Table S1 The preparation method and composition of nutrient solution used in the experiment.

| Treatment (NH_4_^+^/NO_3_^-^) | N source  (mmol L^-1^) | Ca^2+^  (CaCl_2_, mmol L^-1^) | Others macronutrients  (mmol L^-1^) | Fe-EDTA^a^  (μmol L^-1^) | Microelements^b^  (μmol L^-1^) |
| --- | --- | --- | --- | --- | --- |
| N_a_  (100/0) | 6 (NH_4_Cl) | 4 | KCl: 5  KH_2_PO_4_: 1 MgSO_4_: 4 | 50 | H_3_BO_3_: 0.5; MnSO_4_: 0.74; ZnSO_4_: 0.27; CuSO_4_: 0.001; CoCl_2_: 0.001; Na_2_MoO_4_: 0.005;  KI: 0.025 |
| N_r1_  (75/25) | 0.75 [Ca(NO_3_)_2_];  4.5 (NH_4_Cl) | 3.25 |  |  |  |
| N_r2_  (50/50) | 3 (NH_4_NO_3_) | 4 |  |  |  |
| N_r3_  (25/75) | 2.25[Ca(NO_3_)_2_];  1.5 (NH_4_Cl) | 1.75 |  |  |  |
| N_n_  (0/100) | 3 [Ca(NO_3_)_2_] | 1 |  |  |  |

a, Fe-EDTA solution: Dissolve 2.78 g FeSO_4_·7H_2_O and 3.73 g Na_2_EDTA in distilled water and make up the volume to 500 ml. Dissolve 2.5 ml the final solution in 1000 ml nutrient solution.

b, Mother liquor: dissolve 2.23 g MnSO_4_, 0.86 g ZnSO_4_, 2.5 mg CuSO_4_, 25 mg Na_2_MoO_4_·2H_2_O, 2.5 mg CoCl_2_, 0.62 g H_3_BO_3_, and 83 mg KI in 900 ml distilled water and make up the volume to 1000 ml. Dissolve 10 ml mother liquor in 900 ml distilled water and make up the volume to the final 1000 ml. Dissolve 5 ml the final solution in 1000 ml nutrient solution.
